# Multiple comparisons permutation test for image based data mining in radiotherapy

**DOI:** 10.1186/1748-717X-8-293

**Published:** 2013-12-23

**Authors:** Chun Chen, Marnix Witte, Wilma Heemsbergen, Marcel van Herk

**Affiliations:** 1 Department of Radiation Oncology, The Netherlands Cancer Institute, Plesmanlaan 121, 1066CX, Amsterdam, The Netherlands

## Abstract

Comparing incidental dose distributions (i.e. images) of patients with different outcomes is a straightforward way to explore dose-response hypotheses in radiotherapy. In this paper, we introduced a permutation test that compares images, such as dose distributions from radiotherapy, while tackling the multiple comparisons problem. A test statistic *T*_max_ was proposed that summarizes the differences between the images into a single value and a permutation procedure was employed to compute the adjusted p-value. We demonstrated the method in two retrospective studies: a prostate study that relates 3D dose distributions to failure, and an esophagus study that relates 2D surface dose distributions of the esophagus to acute esophagus toxicity. As a result, we were able to identify suspicious regions that are significantly associated with failure (prostate study) or toxicity (esophagus study). Permutation testing allows direct comparison of images from different patient categories and is a useful tool for data mining in radiotherapy.

## Introduction

When planning a radiotherapy treatment, a compromise is made between coverage of the target and exposure of Organs At Risk (OAR). While the dose to the designated target is generally uniform and homogeneous between patients, the dose to surrounding structures can be highly variable, depending on patient geometries, tumor locations, and treatment techniques. Such heterogeneous incidental dose distributions in patients might “accidentally” lead to different treatment outcomes regarding tumor control (e.g. if subclinical disease is important) or normal tissue toxicity. Therefore, applying data mining techniques to incidental dose distributions gives the possibility to explore dose patterns that are associated with clinical outcomes.

The purpose of introducing data mining in radiotherapy is to explore hypotheses for dose-response relationships. In cancer radiotherapy treatment, variations in stem cells, tumor microscopic disease and radiosensitivity distributions can be expected to affect dose-response relationships. Unfortunately, many of them are unknown. Data mining on incidental dose may yield suspicious anatomical features from which –based on biological or clinical considerations– hypotheses for dose-response relationships can be formulated. If validated, such dose-response relationships would eventually provide evidence for better treatment planning, such as refined knowledge of the clinical target volume (CTV), optimal dose painting inside the GTV and more effective sparing of OARs.

Several studies have focused on exploring dose-response relationships from a different perspective than the conventional dose volume histogram (DVH) based method [1-3]. These methods include either exploring the characteristics of dose distributions (e.g. eccentricity, homogeneity), or applying an advanced classifier (e.g. neural network). However, these methods are not easily applicable in the situation where the hypothesized region is *a priori* not known. Directly comparing dose distributions is then a straightforward method of exploring dose-response relationships. Since the dose at each voxel is compared without prior anatomical or geometrical based hypothesis, a voxel-by-voxel based testing is suitable for hypothesis generation, i.e., to localize suspicious regions. In a prostate study [4], the 3D dose prescribed to prostate patients were registered to an anatomy grid and tested voxel-by-voxel (t-test) for relations with failure. Results indicate that a cluster of voxels outside the prostate yield a p-value of less than 0.05. However, obtaining a p-value at every voxel is not yet the complete result. Since a large number of voxels were tested simultaneously, it is likely that the null hypothesis was incorrectly rejected at some voxels (type I errors), and this is the so called multiple comparisons problem.

The aim of this paper is to introduce a multiple comparisons permutation test to compare images between patients in radiotherapy studies. We begin with describing the methodology. Afterwards, we demonstrate the validity of this method with simulations. Finally, we give two examples of applying permutation test in radiotherapy: one study that relates dose to failure for prostate cancer patients [4] and another study that relates dose to acute esophagus toxicity for non-small cell lung cancer (NSCLC) patients.

## Materials and methods

A permutation test involves five steps: 1) register images from different patients, 2) form a null hypothesis, 3) define a scalar test statistic, 4) generate random samples by permuting the true labels of the patients and extract the test statistic from each random sample, 5) calculate the adjusted p-value from the distribution of the test statistic. Thus, instead of a p-value for every voxel, this test gives a single p-value to describe the difference between two imaging datasets.

Suppose we observe a sample of patients with two outcomes: non-event (N) and event (E). These patients are considered to be representative for the entire population. To compare the dose distributions between the two groups, the first step is to register the dose distributions of all patients into the same grid, through an image registration method [5,6]. The null hypothesis then states that there be no difference in dose distributions between the N and E labeled groups. In the following part, we introduce a test statistic *T*_max_, and describe the permutation procedure to compute the adjusted p-value.

### Test statistic

In order to compare the dose distribution for a sample *i* (randomly drawn from the study population) that includes two outcome groups N and E, the most straightforward way is to compute their average dose difference at each voxel, resulting in a dose difference map. To account for multiple comparisons, we can choose the maximum value of such a dose difference map as a single number test statistic. However, the maximum will not be consistent over all random samples (e.g. *i* = 1,…,1000), because it is highly sensitive to the variation or standard deviation (SD) of the dose difference at each voxel over all random samples. For instance, if voxel 1 has an average dose difference of 10 Gy in sample *i* but the SD of the dose difference is 10 Gy over all samples, while voxel 2 has an average dose difference of 8 Gy in sample *i* and the SD of the dose difference is 1 Gy over all samples, the maximum dose difference for sample *i* would be 10 Gy as derived from voxel 1. In fact, it is more likely that in sample *i*, the large 10 Gy dose difference for voxel 1 is due to chance, because it has a large variation over all samples. To account for this effect, the average dose difference *d*_*i*,*k*_ for sample *i*,(*i* = 1,…,*N*_p_) between the E and N groups at voxel *k*,(*k* = 1,…,*N*_v_) should be normalized into *T*_*i*,*k*_ according to an estimate of its SD:

(1) $$\begin{array}{l} d_{i,k}=\mu_{E,i,k}-\mu_{N,i,k} \end{array}$$

(2) $$\begin{array}{l} T_{i,k}=\frac{d_{i,k}}{\sigma_{k}} \end{array}$$

where *μ*_E,*i*,*k*_ and *μ*_N,*i*,*k*_ are the average dose at voxel *k* for group E and N in sample *i*, and *σ*_*k*_ is the standard deviation of *d*_*i*,*k*_ over *N*_p_ samples. *σ*_*k*_ is computed over the random samples generated from the permutation procedure, as described in the following part. As a result, we obtain a normalized dose difference map (or *T*_*i*,*k*_ map) for sample *i*. The test statistic *T*_max,*i*_ is then selected as the maximum value of the *T*_*i*,*k*_ map. Unlike a voxel-by-voxel based test, *T*_max,*i*_ gives a single number that summarizes the discrepancy of the dose distributions between the two label groups, rather than the discrepancy of a particular voxel. Therefore, *T*_max_ accounts for multiple comparisons. Clearly, *T*_max_ is not the only option for extracting a single value test statistic from the *T*_*k*_ map. Other test statistics like the x percentile, are also eligible [7]. However, *T*_max_ is often chosen for its strong control over Type 1 errors [8].

### Permutation test

We then introduce a permutation procedure that generates random samples under the null hypothesis, such that the distribution of *T*_max,*i*_ is determined. A permutation test relies on the rearrangement of the outcome labels. Under the null hypothesis that there is no significant dose difference between group E and N, labels E and N are exchangeable. Thus randomly permuting the labels of the observed sample gives a new randomized sample. For example, in a sample of 5 patients with 3 N and 2 E labels, there are 10 possible label sets, as shown in Table 1. If the first case is the true labelling that we observed, the other 9 labellings are assigned by permutation. In practice, the numbers of both E and N labels are large in the sample, leading to an extremely large and unfeasible number of possible permutations. However, it has been shown in [9] that when the number of patients in the observed sample is large, 1000 permutations can effectively approximate the distribution for the null hypothesis. The permutation test contains the following steps: For every permuted sample *i*, a *d*_*i*,*k*_ is computed for every voxel *k*. After 1000 permutations, the standard deviation *σ*_*k*_ of {*d*_*i*,*k*_,*i* = 1,…,1000} at voxel *k* is computed and the normalized dose difference maps *T*_*i*,*k*_ and ${\tilde{T}}_{k}$ are generated for every permuted sample *i* and the observed sample, respectively. Subsequently, the maximum *T*_max,*i*_ of *T*_*i*,*k*_ is extracted for every permuted sample *i* and the maximum ${\tilde{T}}_{\text{max}}$ of ${\tilde{T}}_{k}$ is extracted from the observed sample. Thus, after 1000 permutations, we obtain a distribution of *T*_max,*i*_ under the null hypothesis. Finally, the adjusted p-value is computed as the proportion of the permutation samples that yield a higher *T*_max_ value than the ${\tilde{T}}_{\text{max}}$ observed with the true labels. If the adjusted p-value is smaller than significance level *α* (e.g. 0.05), we reject the null hypothesis. Furthermore, the (1-*α*) percentile of the *T*_max,*i*_ distribution gives a threshold value *T*^∗^. In the observed normalized dose difference map ${\tilde{T}}_{k}$, voxels higher than *T*^∗^ show significant dose difference between E and N groups. The mathematical formulation of the permutation test is presented in more detail in Appendix A: the multiple comparisons permutation test.

**Table 1:** The 10 possible label combinations of 3 Ns and 2 Es

1. N N N E E	6. N E E N N
2. N N E N E	7. E N N N E
3. N N E E N	8. E N N E N
4. N E N N E	9. E N E N N
5. N E N E N	10. E E N N N

If the first case is the true labels that we observed, the other 9 labels are assigned by permutation.

### Simulation

**Figure 1:**
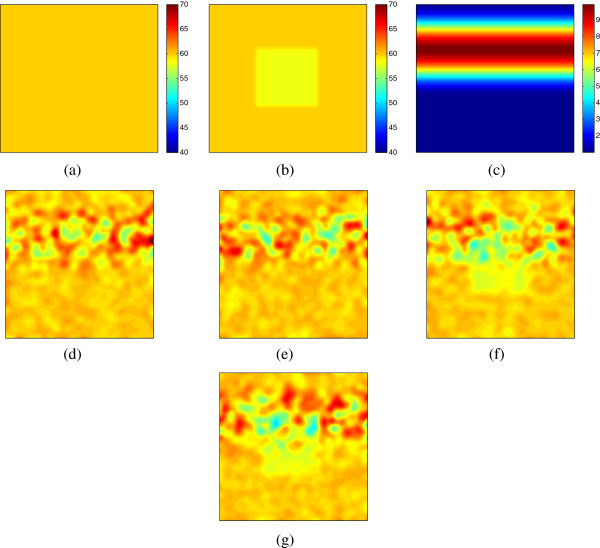
**Simulated dose images (128 × 128 pixels) in two groups (E and N).** **(a)** and **(b)** are the average dose map for E and N groups. **(c)** is the simulated SD of variations. Each group was simulated with 50 images. **(d-e)** and **(f-g)** are two examples of the dose map for group E and N, respectively.

To demonstrate the permutation test, we conducted simulations on two groups of artificially generated dose images (with 128 × 128 pixels). Each group had 50 images. As seen in Figure 1(a) and 1(b), group E had a homogeneous average dose of 60 Gy, while for group N, a block (50 × 50) of average 58 Gy was located inside the dose image. At each pixel, we simulated the additive dose variation as a normal distribution *N* ∼ (0,*σ*). Figure 1(c) illustrates the simulated values for *σ*. Large variations were simulated in the upper part (*σ*_max_ = 10 Gy), while small variations were in the lower part (*σ*_min_ = 1 Gy). After adding noise to the average dose, a smooth Gaussian filter was applied to each patient’s dose image. The purpose of generating spatially correlated variation and applying Gaussian filter is to mimic actual spatially correlated incidental dose in planning. We first illustrate how the the multiple comparison permutation test works step-by-step. Afterwards, we conduct a sensitivity analysis to show how the result changes according to the average dose difference and the sample size, compared to the voxel-by-voxel based t-test.

**Figure 2:**
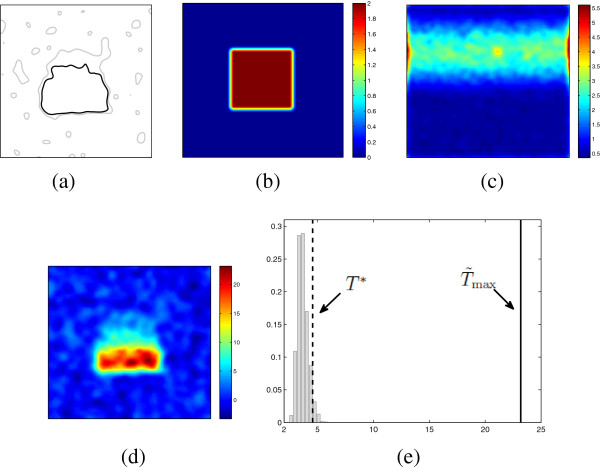
**Permutation test procedure with simulations.** **(a)** The identified region with *p* < 0.05, through a voxel-by-voxel based t-test (gray contour) and the permutation test (black contour). **(b)** The average dose difference map ${\tilde{d}}_{k}$ (*k* = 1,…,128×128) for the observed sample. **(c)** The SD *σ*_*k*_ of the dose difference, computed from 1000 permutated random samples. **(d)** The normalized dose difference map ${\tilde{T}}_{k}$ for the observed sample, which is **(b)** divided by **(c)**. **(e)** The distribution of *T*_max,*i*_ from *i* = 1,…,1000 permutation samples, where ${\tilde{T}}_{\text{max}}$ is the maximum value in figure **(d)** and *T*^∗^ is the 95 percentile of *T*_max,*i*_.

First, a voxel-by-voxel based t-test was applied to these two groups. Pixels obtaining a *p* < 0.05 are illustrated in Figure 2(a), showing a big central region as well as many isolated spots. These isolated spots obtained a low p-value by chance. For the permutation test, Figures 2(b)– 2(d) show the average dose difference map for the observed sample, the SD of the average dose difference from 1000 permutation random samples, and the consequent normalized average dose difference map for the observed sample, respectively. The distribution of *T*_max,*i*_ from 1000 random samples is illustrated in Figure 2(e). The ${\tilde{T}}_{\text{max}}$ from the observed sample is much larger than any of the *T*_max,*i*_, thus the adjusted p-value is zero, i.e. there is a highly significant dose difference between the two groups. The 95% of the *T*_max,*i*_ distribution gives a threshold *T*^∗^, by applying it to the ${\tilde{T}}_{k}$ map, we obtained the second region illustrated in Figure 2(a). This region is smaller than that from a voxel-by-voxel based test. Additionally, the isolated spots, which would probably falsely reject the null hypothesis by the voxel-by-voxel based test, are excluded. Therefore, compared to the voxel-by-voxel based test, permutation test provides a statistically stronger result, in terms of an adjusted p-value and a more accurate region with dose differences.

**Figure 3:**
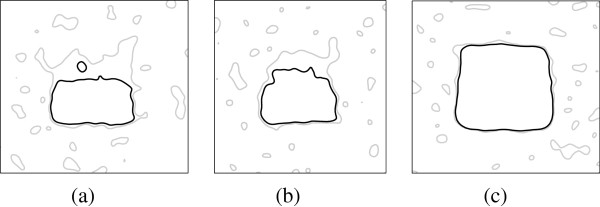
**The identified region with *p*** **< 0.05, through a voxel-by-voxel based t-test (gray contour) and the permutation test (black contour), given the average dose difference of (a) 0.5 Gy, (b) 2 Gy and (c) 10 Gy.**

Furthermore, Figure 3 shows the simulation results by increasing the average dose difference, from 0.5 Gy, 2 Gy (example above) to 10 Gy, given the other parameters fixed as the above example. The larger the average dose difference is, the more regions were detected for both multiple comparisons test and t-test, especially at the top of the square region, where the dose variance is higher (Figure 2(c)). Multiple comparison test becomes more conservative than the t-test, when the average dose difference is small. However, t-test always ends up with more isolated false positive spots (type-I error).

**Figure 4:**
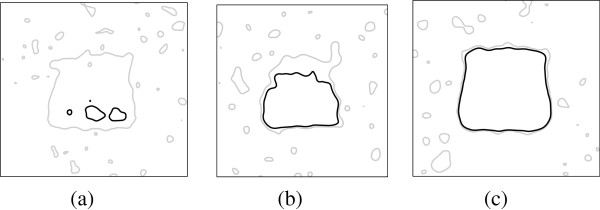
**The identified region with *p*** **< 0.05, through a voxel-by-voxel based t-test (gray contour) and the permutation test (black contour), given the number of patients of (a) n = 5, (b) n = 50 and (c) n = 100.**

Similarly, Figure 4 shows the simulation results by increasing the number of patients, from *n* = 5, *n* = 50 to *n* = 100. Results show that multiple comparison test becomes more conservative than the t-test, especially when the sample size is small. However, in any cases, t-test ends up with more isolated false positive spots (type-I error).

### Applications

#### Study I: prostate

We applied the permutation test on data used by [4]. The aim of this study was to relate dose distributions with failure in prostate cancer patients. We selected a group of 67 patients with a relatively higher risk for extraprostatic disease, estimated according to T-stage, iPSA and Gleason score or differentiation grade [10]. These patients were treated in Erasmus Medical Center, The Netherlands, and they were included in the Dutch Phase III trial (CKVO 96-10) with dose randomized between 68 Gy and 78 Gy [11]. The Ethical Committee of each institution approved the protocol. Patients mainly had tumors of stage T3b and were treated to the delineated prostate and the seminal vesicles. The extra boost of 10 Gy had a 5 mm margin to the CTV (except towards the rectum, where a 0 mm margin was applied). For the 68 Gy PTV, a 10 mm margin was applied. In this study, the failure was biochemical (PSA nadir plus 2) [12] or clinical (locoregional or distant progression or start of salvage hormone therapy), determined at a fixed 4 year endpoint. As a result, 37 failure patients and 30 non-failure patients were eligible for analysis. Delineations from the planning CT and the planned dose distributions were collected for each patient. Firstly, dose distributions of all patients were registered into a dose grid as described in [4]. In short, voxels correspond if their direction with respect to the center of mass (CM) is the same, and their distance to the surface in this direction is the same. For voxels inside the prostate, corresponding voxels have the same fractional distance between the CM and the surface. The registration identifies anatomical points at locations relative to the delineated prostate surface. The choice for this registration procedure is an important part of the dose-effect hypothesis, and was based on the suspicion that extracapsular extension might have affected outcome. The resulting grid has a dimension of 31 × 35 × 34, resulting in *N*_v_ = 36890 dose voxels. The null hypothesis is that there is no dose distribution difference between the failure and the non-failure patients. The multiple comparison permutation test was applied to the registered dose maps.

#### Study II: esophagus

We applied the permutation test on a esophagus toxicity study. The aim of this study is to relate dose distributions on the esophagus surface with acute esophagus toxicity (AET) in NSCLC patients. We selected 185 NSCLC patients treated in Netherlands Cancer Institute (NKI) from 2008 to 2010 with concurrent chemotherapy combined with IMRT. The RT dose was 66 Gy in 24 fractions. The concurrent chemotherapy included a daily low dose cisplatin [13]. AET was scored according to the Common Toxicity Criteria 3.0. Toxicity was scored weekly from baseline until 3 weeks after RT. Afterwards, patients were checked every 2 months or more frequently if necessary. Of the 185 patients, 76 had no or grade 1 AET; 67 patients developed grade 2 and 42 patients had grade 3; Grade 4 or 5 AET did not occur. The delineated esophagus from the planning CT and the planned dose distributions were retrospectively collected for each patient, allowing a 2D esophagus surface dose map (ESDM) to be computed. For each patient, dose was sampled on every slice of the CT scan (3 mm thickness) at 36 fixed orientations along the delineated esophagus contour to the center, from 0 to 360 degrees with 10 degrees increment. The 0 degree angle was always chosen in the Right-Left direction. (In our experience the esophagus is always star shaped, i.e. the full contour can always be seen from a single centreline.) The same sampling procedure was then done through all the m slices where the esophagus was delineated. As a result, the ESDM contains *m* × 36 pixels for every patient (*m* varies from patient to patient). ESDMs of all patients were registered such that the pixel with the highest dose is in the center of the 2D dose map, alowing translations along and rotations around the length of the esophagus. The choice for this mapping was based on the assumption that the length and the circumference of the high dose region on the esophagus surface is associated with AET, irrespective of its anatomical location. Permutation tests were applied to find differences between grade 0–1 and grade 2–3, and between grade 0–2 and grade 3 AET.

## Results

### Results of study I

**Figure 5:**
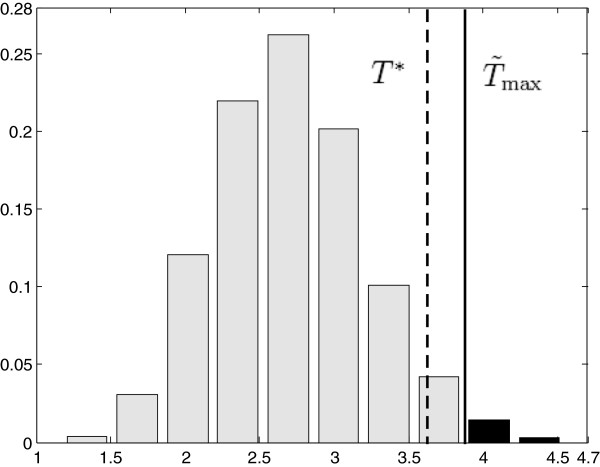
**The histogram of** ***T***_**max,*i***_** obtained from the 1000 random permutations.** The adjusted p-value (*P* = 0.02) is computed as the black area larger than the observed ${\tilde{T}}_{\text{max}}$ (solid line). The 95 percentile *T*^∗^ (dashed line) determines the region with significant dose difference as shown in Figure 6.

**Figure 6:**
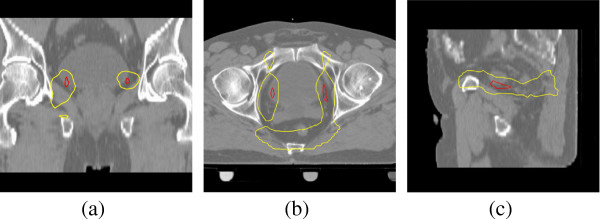
**The region (marked in red) with significant dose difference (adjusted p-value < 0.05) from the permutation test in the prostate study, together with the region (marked in yellow) with p-value < 0.05 in a voxel-by-voxel based t test.** **(a)** coronal view; **(b)** axial view; **(c)** sagittal view.

The distribution of *T*_max,*i*_,(*i* = 1,…,1000) from the 1000 random permutations under the null hypothesis is shown in Figure 5. The proportion of *T*_max,*i*_ that are larger than the observed value ( ${\tilde{T}}_{\text{max}}=3.81$) gives an adjusted p-value of 0.02, i.e., there is a significant dose difference between the non-failure and the failure patients for this patient group at *α* = 0.05 significance level. Furthermore, the 95 percentile of the *T*_max,*i*_ distribution gives the threshold *T*^∗^ = 3.56. In Figure 6, voxels above this threshold in the observed ${\tilde{T}}_{k}$ map are marked, overlaid on the CT scan of the standard anatomy grid. This region is situated in the obturator region and suggests nodal involvement, but does not correspond to the presumed extracapsular extension. The significant region is much smaller than that obtained through a voxel-by-voxel based t test [4]. The adjusted value p = 0.02 was significant for the test statistic *T*_max_. Compared to the previous voxel-by-voxel based testing method, the permutation test gives a statistically stronger conclusion that there is indeed a difference in the dose distribution outside the prostate between failure and non-failure patients.

### Results of study II

**Figure 7:**
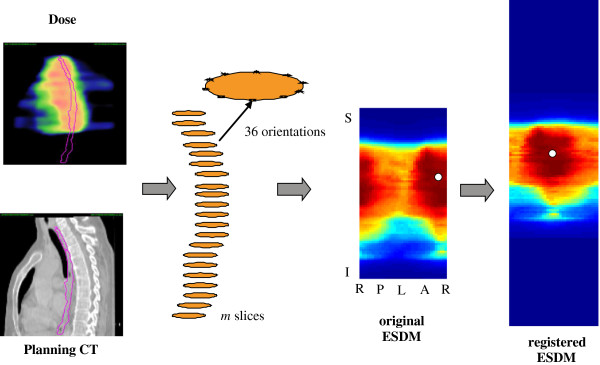
**An example of generating an esophagus surface dose map (ESDM).** The pixel with the highest dose (in white) is registered to the center location in the registered ESDM through translation and rotation of the original ESDM.

**Figure 8:**
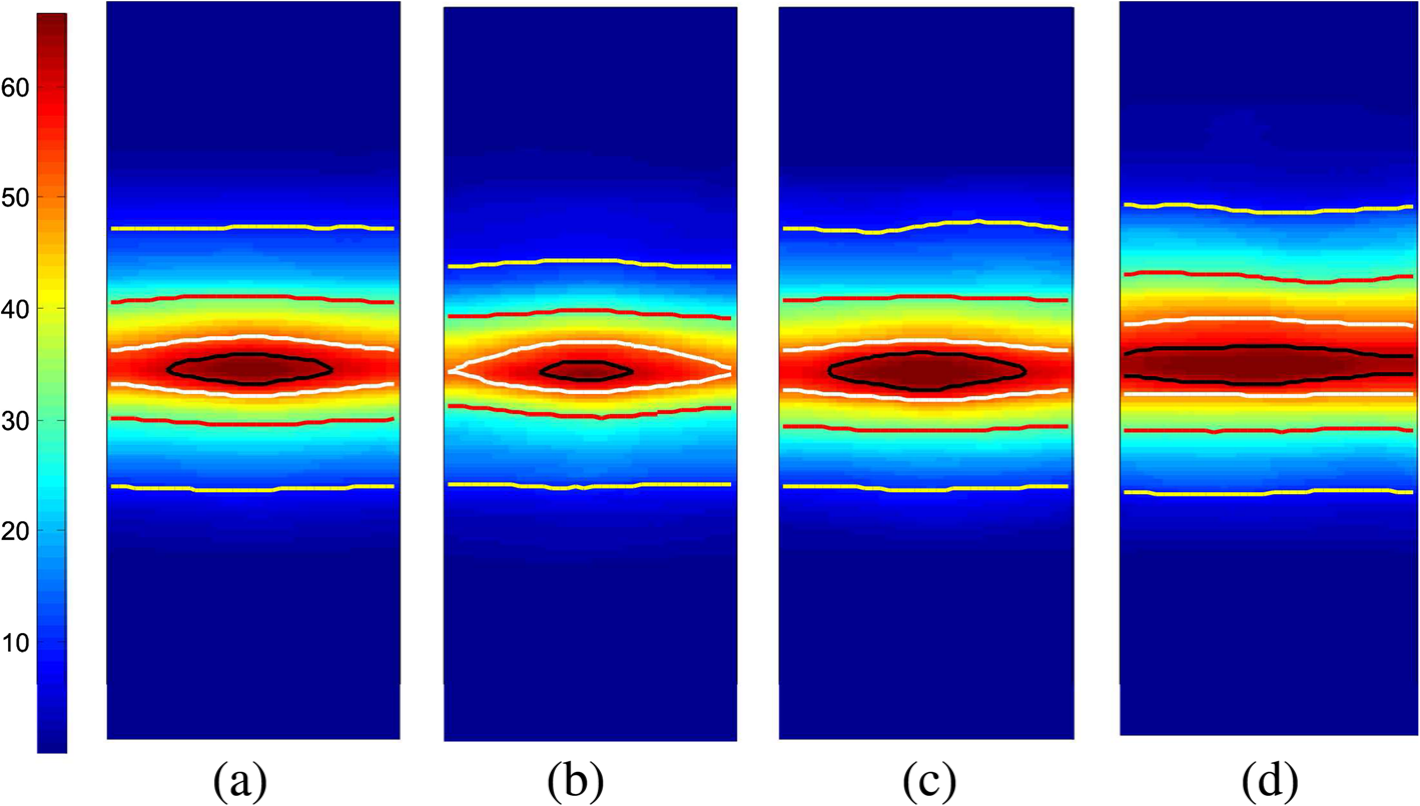
**The average dose map of the locally registered ESDMs.** **(a)** all patients; **(b)** patients with AET Grade 0–1; **(c)** patients with AET Grade 2; **(d)** patients with AET Grade 3. The iso-dose lines of 60 Gy, 50 Gy, 30 Gy and 10 Gy are marked in black, white, red and yellow.

An example of generating an ESDM is illustrated in Figure 7. The average dose maps of the registered ESDMs for all 185 patients as well as each toxicity grade subgroup are illustrated in Figure 8. The differences in the isodose lines for patients with grade 0–1 until grade 3 AET imply that for patients of increasing AET grade, their average esophagus surface that received high dose increases: All isodose lines expand along the length of the esophagus for patients with more complications. Specifically, the 50 Gy and 60 Gy isodose lines are expanding through both the length and the circumference.

**Figure 9:**
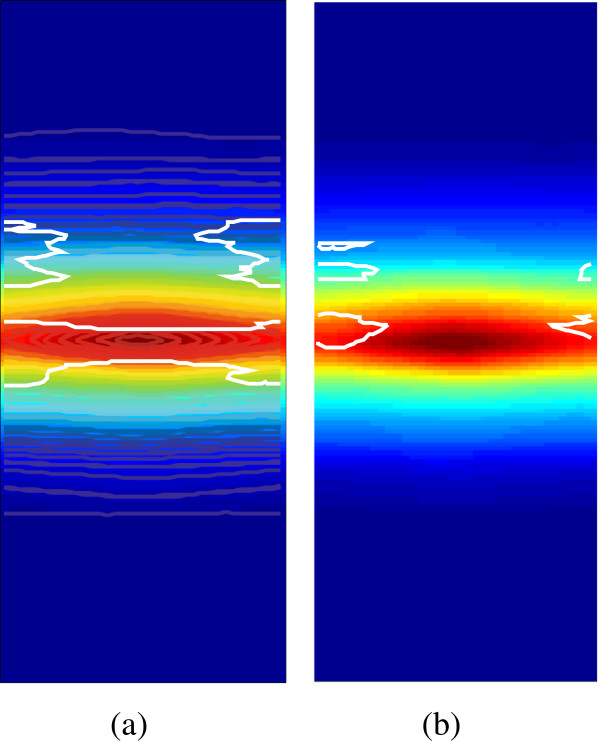
The region (in white contour) with significant dose difference (a) between AET Grade 0–1 and 2–3, and (b) between AET Grade 0–2 and 3, overlaid on the average dose map of the total 185 patients.

The *T*_max_ distribution, adjusted p-value and *T*^∗^ threshold were computed in the same way as in study 1. The adjusted p-value for the dose distribution between patients with AET grade 0–1 and grade 2–3 is *p* < 0.001, showing a significant dose difference at *α* = 0.05 significance level. The region with significant dose difference is illustrated in Figure 9(a). Note that the region refers to a certain shape of dose distribution (as the highest doses were mapped to the same point), rather than a specific anatomical area on the esophagus. Considering the esophagus as a tube, this region includes a full band covering the high dose area (35 to 65 Gy) and a sub-band covering the moderate dose region (10 to 30 Gy). Similarly, the adjusted p-value for the dose distribution of patients between AET grade 0–2 and grade 3 is *p* = 0.002. The region with significant dose difference is illustrated in Figure 9(b), it appears to be part of the significant region in Figure 9(a), showing a high dose (50 to 60 Gy) area with extra length and circumference coverage.

## Discussion

In this paper, we introduced multiple comparison permutation testing for voxel based data mining in radiotherapy and we demonstrated the test in two studies. For both studies we were able to locate regions where dose significantly associates with the outcome. In the prostate study, we were able to provide strong statistical evidence for a dose difference between non-failure and failure patients, confirming a difference located in the obturator region that could be suspicious for subclinical disease. In the esophagus study, both regions to predict grade ≥2 and grade 3 are consistent with the V50 dose volume histogram (DVH) parameter as derived in study [13]. Grade 2 seems to be caused by high dose (≥50 Gy) and the length/circumference coverage of low dose, while the length/circumference coverage of high dose (around 50 Gy) plays a role in severe AET of grade 3. This result suggests that using the length and circumference parameters may be a more sensitive method to predict AET compared to DSHs.

A broadly recognized method to address the problem of multiple comparisons is the Bonferroni correction [14]: if n independent hypotheses are tested, each individual hypothesis is tested at the 1/n times of the original statistical significance level when tested for only one hypothesis. However, this correction is not straightforwardly applicable to voxel maps, since there can be millions of voxels that are highly correlated in space. Hypothesis testing on images was first conducted through parametric random field methods [15]: t-tests are conducted at every voxel and the distributional results for continuous random fields are used to identify regions that are significant. Contrary to these parametric methods, non-parametric permutation tests on voxel maps were introduced by [8,16]. Two test statistics are often used: a single maxima threshold and the supra-threshold cluster. A single maxima threshold is the *T*_max_ as we used in our study. In [16], *T*_max_ was applied in a permutation test to localize the region of visual cortex sensitive to motion on 3D PET imaging [17] and to analyze the order effects in working memory using fMRI [18]. Contrarily, a supra-threshold cluster test assesses the size of the connected supra-threshold regions for significance. As a result, the power to localize regions was reduced. Since the goal of data mining in radiotherapy was to localize suspicious regions, we recommend using *T*_max_ as test statistic.

The incidental dose essentially comes from the variations of dose planning for some un-targeted organs, and it’s a good thing to explore. Whether or not we are able to detect a significant dose difference depends on two aspects: 1) the average incidental dose between 2 groups and 2) the variance of the incidental dose. Statistically, a higher average incidental dose difference and a lower variance of each group facilitates yielding a true positive result. On the other hand, registration error is a bad thing. Inaccuracies in the registration, or an inappropriate choice for the registration method, could prevent the method to identify an existing dose effect relation, thus reducing the power of the statistical test. While it seems less likely that some particular registration procedure or inaccuracy therein generates a false positive result from dose variations which only consist of noise, any dose effect relation which is subsequently derived should be verified on an independent data set. Depending on the specific anatomical properties and the expected dose-effect parameters, the requirements for the accuracy of the registration vary. For instance, if the data mining is conducted in regions with small structures (e.g. head and neck), a sophisticated registration procedure may be required to find significant results. Contrarily, if we want to explore a large volume of low gradient dose distributions (e.g. lung), a loose registration may suffice. If we aim to explore dose distributions surrounding one structure, the registration accuracy is then focused on regions close to this structure. Therefore, a registration strategy should be chosen in advance based on the type of hypothesis that we want to explore. Afterwards, significant regions can be anatomically identified, and subjected to biological and clinical interpretation. Such consideration can then guide further efforts to derive dose-response relationships through conventional modeling methods.

Permutation testing is a useful tool to explore dose patterns from incidental dose distributions. Instead of analyzing dose-response effect, we intend to use permutation testing as a preliminary step to identify suspicious regions for hypothesis generation. Permutation testing takes into account multiple comparisons by yielding an adjusted p-value and gives visually straightforward suspicious regions. Another advantage of such method is that it is non-parametric. Thus, this test does not depend on the assumption of Normal distribution, which is often not true in the case of incidental dose in the planning. Permutation testing is practically useful and important in radiotherapy, especially in the era where adaptive radiotherapy is on the agenda, but we still have only limited knowledge about tumor stem cells, microscopic disease, radiosensitivity, etc. Permutation testing helps us to maximally explore dose-response relationships from the incidental dose in the clinical data.

## Conclusions

We introduced a permutation test that deals with hypothesis testing on images and illustrated this method in a synthetic dataset, and in clinical datasets from a prostate and an esophagus study. Compared to a voxel-by-voxel based test, the permutation method reduces the rate of false positives. Permutation testing is a useful tool to identify hypotheses for dose-response relationships and tackle the multiple comparisons problem.

## Consent

Written informed consent was obtained from the patient for the publication of this report and any accompanying images.

## Appendix A: the multiple comparisons permutation test

Suppose we observed a sample of patients with two outcomes: non-event (N) and event (E). Every patient has a dose distribution of *N*_v_ voxels and they are all registered to an identical grid. To compare the dose distribution between the two groups, the permutation test is conducted as follows: 

(i) Compute the average dose difference $\tilde{d_{k}}$ between E and N groups in the observed sample:

(A.1) $$\tilde{d_{k}}={\tilde{\mu }}_{E,k}-{\tilde{\mu }}_{N,k},k=1,\ldots ,N_{v},$$

where ${\tilde{\mu }}_{E,k}$ and ${\tilde{\mu }}_{N,k}$ are the average dose value at voxel *k* for group E and N, respectively.

(ii) Permute the labelling of the observed sample and compute the average dose difference. Repeat this process for *N*_p_ times:

(A.2) $$d_{i,k}=\mu_{E,i,k}-\mu_{N,i,k},k=1,\ldots ,N_{v},i=1,\ldots ,N_{p},$$

where *μ*_E,*i*,*k*_ and *μ*_N,*i*,*k*_ are the average dose value at voxel *k* for group E and N in the *i*^th^ permuted random sample.

(iii) Compute the standard deviation for every voxel *k* over all *N*_p_ random samples:

(A.3) $$\sigma_{k}=\sqrt{\frac{1}{N_{p}-1}\overset{N_{p}}{\underset{i=1}{\sum }}(d_{i,k}-{\bar{d}}_{k})},\;\text{where}\;{\bar{d}}_{k}=\frac{1}{N}\overset{N_{p}}{\underset{i=1}{\sum }}d_{i,k}.$$

(iv) Compute the locally normalized dose difference for every voxel in every random sample as well as the observed sample:

(A.4) $$\begin{array}{l} T_{i,k}=\frac{d_{i,k}}{\sigma_{k}}, \end{array}$$

(A.5) $$\begin{array}{l} {\tilde{T}}_{k}=\frac{{\tilde{d}}_{k}}{\sigma_{k}},k=1,\ldots ,N_{v}. \end{array}$$

(v) Compute the test statistic *T*_max_ for every resampling as well as the true labeling sample:

(A.6) $$\begin{array}{l} T_{\text{max},i}=\max(T_{i,k}), \end{array}$$

(A.7) $$\begin{array}{l} {\tilde{T}}_{\text{max}}=\max({\tilde{T}}_{k}),k=1,\ldots ,N_{v}. \end{array}$$

(vi) Compute the adjusted p-value:

(A.8) $$p=\text{Pr}\left[T_{\text{max},i}>{\tilde{T}}_{\text{max}}\right].$$

(vii) Compare the adjusted p-value with the significance level *α*. If *p* < *α*, reject the null hypothesis, otherwise the null hypothesis can not be rejected.

(viii) Compute the *T*^∗^ as (1 - *α*) percentile of *T*_max_,*i* = 1,…,*N*_p_. Regions in ${\tilde{T}}_{k}$ that are above *T*^∗^ show significant difference between E and N groups.

## Competing interests

The authors declare that they have no competing interests.

## Authors’ contributions

All authors participated in the study and drafted the manuscript. All authors read and approved the final manuscript.
